# Comparison of Maternal Labor-Related Complications and Neonatal Outcomes Following Elective Induction of Labor at 39 Weeks of Gestation vs Expectant Management: A Systematic Review and Meta-analysis

**DOI:** 10.1001/jamanetworkopen.2023.13162

**Published:** 2023-05-12

**Authors:** James Hong, Jessica Atkinson, Alexandra Roddy Mitchell, Stephen Tong, Susan P. Walker, Anna Middleton, Anthea Lindquist, Roxanne Hastie

**Affiliations:** 1 Department of Obstetrics and Gynaecology, University of Melbourne, Heidelberg, Victoria, Australia; 2 Mercy Perinatal, Mercy Hospital for Women, Heidelberg, Victoria, Australia

## Abstract

**Importance:**

Elective induction of labor at 39 weeks of gestation is common. Thus, there is a need to assess maternal labor-related complications and neonatal outcomes associated with elective induction of labor.

**Objective:**

To examine maternal labor-related complications and neonatal outcomes following elective induction of labor at 39 weeks compared with expectant management.

**Data Sources:**

A systematic review of the literature was conducted using the MEDLINE (Ovid), Embase (Ovid), Cochrane Central Library, World Health Organization, and ClinicalTrials.gov databases and registries to search for articles published between database inception and December 8, 2022.

**Study Selection:**

This systematic review and meta-analysis included randomized clinical trials, cohort studies, and cross-sectional studies reporting perinatal outcomes following induction of labor at 39 weeks vs expectant management.

**Data Extraction and Synthesis:**

Two reviewers independently assessed study eligibility, extracted data, and assessed studies for bias. Pooled odds ratios (ORs) and 95% CIs were calculated using a random-effects model. This study is reported per the Preferred Reporting Items for Systematic Reviews and Meta-analyses 2020 guideline, and the protocol was prospectively registered with PROSPERO.

**Main Outcomes and Measures:**

Maternal outcomes of interest included emergency cesarean section, perineal injury, postpartum hemorrhage, and operative vaginal birth. Neonatal outcomes of interest included admission to the neonatal intensive care unit, low 5-minute Apgar score (<7) after birth, macrosomia, and shoulder dystocia.

**Results:**

Of the 5827 records identified in the search, 14 studies were eligible for inclusion in this review. These studies reported outcomes for 1 625 899 women birthing a singleton pregnancy. Induction of labor at 39 weeks of gestation was associated with a 37% reduced likelihood of third- or fourth-degree perineal injury (OR, 0.63 [95% CI, 0.49-0.81]), in addition to reductions in operative vaginal birth (OR, 0.87 [95% CI, 0.79-0.97]), macrosomia (OR, 0.66 [95% CI, 0.48-0.91]), and low 5-minute Apgar score (OR, 0.62 [95% CI, 0.40-0.96]). Results were similar when confined to multiparous women only, with the addition of a substantial reduction in the likelihood of emergency cesarean section (OR, 0.61 [95% CI, 0.38-0.98]) and no difference in operative vaginal birth (OR, 1.01 [95% CI, 0.84-1.21]). However, among nulliparous women only, induction of labor was associated with an increased likelihood of shoulder dystocia (OR, 1.22 [95% CI, 1.02-1.46]) compared with expectant management.

**Conclusions and Relevance:**

In this study, induction of labor at 39 weeks was associated with improved maternal labor-related and neonatal outcomes. However, among nulliparous women, induction of labor was associated with shoulder dystocia. These results suggest that elective induction of labor at 39 weeks may be safe and beneficial for some women; however, potential risks should be discussed with nulliparous women.

## Key Points

**Question:**

What maternal labor-related and neonatal outcomes are experienced following elective induction of labor at 39 weeks of gestation compared with expectant management?

**Findings:**

In this systematic review and meta-analysis of 14 studies with more than 1.6 million participants, induction of labor at 39 weeks of gestation was associated with improved maternal labor-related and neonatal complications, including a reduced likelihood of perineal injury, macrosomia, and low 5-minute Apgar score after birth. However, among nulliparous women only, induction of labor was associated with an increased likelihood of shoulder dystocia compared with expectant management.

**Meaning:**

These findings suggest that elective induction of labor at 39 weeks may be safe and beneficial for some women; however, potential risks should be discussed with nulliparous women.

## Teaser

This systematic review and meta-analysis assesses maternal labor-related and neonatal outcomes associated with elective induction of labor at 39 weeks of gestation compared with expectant management.

## Introduction

Induction of labor is recommended when the maternal and perinatal risks of continuing pregnancy outweigh those associated with expedited birth. Induction of labor may be indicated for postterm pregnancies beyond 41 weeks, in suspected cases of poor fetal growth, or for medical reasons such as prelabor rupture of membranes or hypertension.^1,2^ The benefits of indicated induction of labor have been well characterized.^3,4,5,6^

Elective induction of labor is defined as induction in the absence of any medical indication.^7^ Historically, elective induction has been discouraged due to the associated increased risk of cesarean birth and adverse birth outcomes compared with spontaneous labor.^8,9^ However, this is not an appropriate comparator, given that forgoing elective induction will not always result in spontaneous labor. A more clinically relevant comparator is expectant management, defined as a “watch-and-wait” approach, allowing the pregnancy to continue until labor begins spontaneously or there is a reason to induce later.^1^

Elective induction at term is increasing globally and is likely attributable to the findings of the landmark ARRIVE trial (A Randomized Trial of Induction Versus Expectant Management).^10^ The ARRIVE trial demonstrated that elective induction at 39 weeks of gestation among low-risk nulliparous women is associated with a reduced incidence of cesarean birth, without an increase in adverse perinatal outcomes compared with expectant management.^10^ These findings have been supported by subsequent studies, some even suggesting a clear reduction in perinatal mortality.^11,12^ These studies have led to greater confidence among clinicians about the safety of elective induction at term.^13^ Additionally, our team has identified that developmental outcomes for children born after induction of labor at 39 weeks of gestation do not differ from those of their expectantly managed peers.^14^

While these findings are encouraging, most studies have focused on perinatal outcomes (except for rates of cesarean section and operative birth). There have been very few studies on the impact of induction at 39 weeks on maternal labor-related complications, such as perineal injury and postpartum hemorrhage.^15,16,17^ Additionally, many previous studies have excluded women with a high body mass index (BMI [calculated as weight in kilograms divided by height in meters squared]) or those undergoing a trial of labor after cesarean section. This study aimed to investigate maternal labor-related complications following elective induction at 39 weeks of gestation compared with expectant management and included nulliparous and multiparous women as well as those with a high BMI or those undergoing a trial of labor after cesarean section.

## Methods

### Eligibility, Information Sources, and Search Strategy

We performed a systematic review and meta-analysis to investigate maternal labor-related complications following induction of labor at 39 weeks of gestation compared with expectant management. The primary search terms related to labor, induction of labor, and perinatal outcomes and are provided in eTable 1 in Supplement 1. We searched the MEDLINE (Ovid), Embase (Ovid), Cochrane Central Library, World Health Organization, and ClinicalTrials.gov databases and registries for articles published between database inception and December 8, 2022. Randomized clinical trials, cohort studies, and cross-sectional studies investigating the association between elective induction at 39 weeks and perinatal outcomes were included. The included studies compared individuals with an elective induction of labor at 39 weeks of gestation with those who received expectant management thereafter. Studies were excluded if the induction group included individuals with medical indications for induction, if only multiple pregnancies were assessed, or if gestational age parameters were unclear.

This study was registered with PROSPERO (CRD42020204732) and reported according to Preferred Reporting Items for Systematic Reviews and Meta-analyses (PRISMA) guideline.^18^ This study was exempt from institutional review board approval by the Mercy Health Human Research Ethics Committee and informed patient consent requirements were waived because this was a secondary use of deidentified data sets.

### Study Selection and Data Extraction

Covidence^19^ systematic review software was used for study screening and data extraction. After duplicate studies were removed, 2 reviewers (any 2 of J.H., J.A., A.R.M., or A.M.) independently screened titles, reviewed full texts, and extracted data from eligible studies. Discrepancies were resolved by a third reviewer (R.H.). Where articles reported results from the same study population, results from the larger study were included.^20,21^ The following data were extracted: author, year of publication, country of study, study design, study population, parity, maternal outcomes (emergency cesarean section, obstetric anal sphincter injury, postpartum hemorrhage, and operative vaginal delivery), and neonatal outcomes (admission to the neonatal intensive care unit [NICU], low 5-minute Apgar score [<7] after delivery, macrosomia, and shoulder dystocia).

### Risk-of-Bias Assessment

Risk of bias was assessed by 2 independent reviewers (J.H. and A.R.M.) using the Newcastle-Ottawa Scale (NOS) for nonrandomized studies and the Cochrane Risk of Bias 2 (RoB 2) tool for randomized studies.^22,23^ The NOS examines the quality of nonrandomized studies across 3 domains: study group selection, comparability between the groups, and ascertainment of relevant outcomes (cohort studies) or exposures (case-control studies). The Cochrane RoB 2 tool examines bias across 5 domains: risk of bias arising from randomization, risk of bias due to deviation from intended interventions, missing outcome data, risk of bias in outcome measurement, and risk of bias in reporting results. Conflicts were resolved via reviewer discussion and consultation with an independent third reviewer (R.H.).

### Statistical Analysis 

For studies reporting the same outcomes, outcome data were pooled using a random-effects model. Results are presented as odds ratios (ORs) with corresponding 95% CIs. For pooled estimates, the *I*^2^ statistic was used to quantify heterogeneity. Subgroup analyses by parity (nulliparity and multiparity) were performed. Statistical analysis was performed using StataMP, version 17 (StataCorp LLC).

## Results

### Study Selection

**Figure 1.**
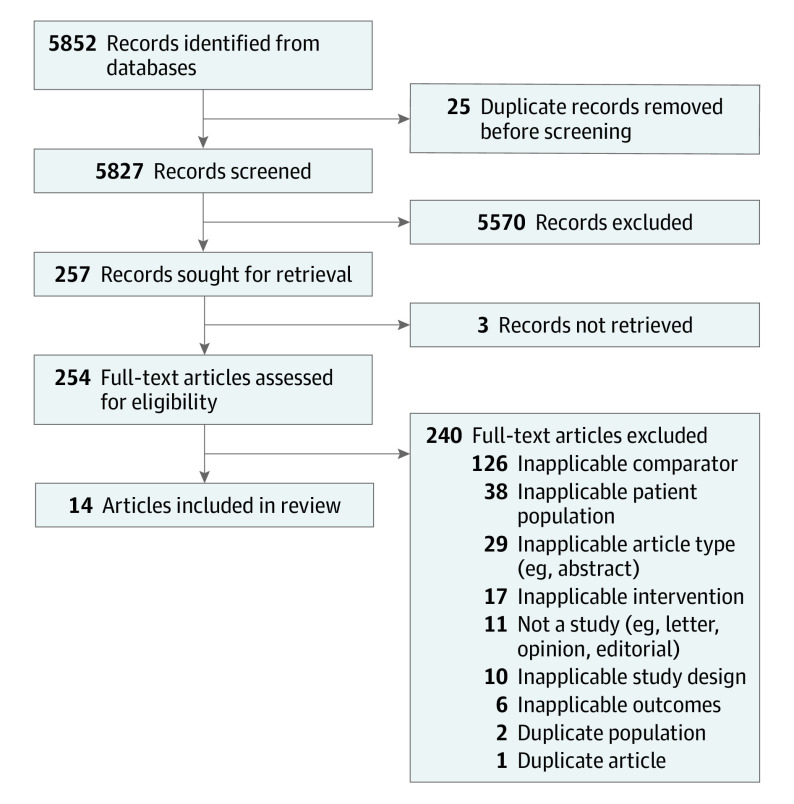
Study Flow Diagram

Our search identified 5827 articles. After title and abstract screening, 254 full-text articles were screened; of these, 14 studies^10,20,21,24,25,26,27,28,29,30,31,32,33,34^ were included (Figure 1). These studies reported outcomes of women with a singleton pregnancy, including 86 555 women who were induced at 39 weeks of gestation. There were 12 retrospective cohort studies,^20,21,24,25,26,28,29,30,31,32,33,34^ 1 cross-sectional study,^27^ and 1 randomized clinical trial^10^ (Table 1).

**Table 1.** Characteristics of Included Studies

Source	Study design	No. of participants	Population
Bailit et al,^24^ 2015	Retrospective cohort	24 027	Nulliparous women with a singleton vertex nonanomalous pregnancy
Cheng et al,^25^ 2012	Retrospective cohort	61 712	Nulliparous women delivering between 39 and 42 weeks of gestation
Darney et al,^26^ 2013	Retrospective cohort	151 707	Women with a singleton pregnancy
Gibbs Pickens et al,^20^ 2018	Retrospective cohort	108 662	Women with a BMI >30 and a singleton pregnancy in cephalic presentation
Gibson et al,^27^ 2014	Cross-sectional	51 600	Women with a singleton pregnancy in vertex presentation
Grobman et al,^10^ 2018	Randomized clinical trial	6096	Low-risk nulliparous women with a nonanomalous singleton pregnancy in vertex presentation
Lappen et al,^28^ 2015	Retrospective cohort	3968	Women with a singleton pregnancy undergoing a trial of labor after a previous cesarean section
Lee et al,^21^ 2016	Retrospective cohort	37 723	Women with a BMI >30 with a singleton pregnancy and no preexisting comorbidities
Palatnik and Kominiarek,^29^ 2020	Retrospective cohort	9375	Women recruited to the consortium safe labor database with a BMI >30 and a singleton pregnancy in cephalic presentation
Park et al,^30^ 2022	Retrospective cohort	50 229	Women with a singleton pregnancy and one prior cesarean section birth
Sinkey et al,^31^ 2019	Retrospective cohort	2626	Low-risk nulliparous women with a nonanomalous singleton pregnancy in vertex presentation
Souter et al,^32^ 2019	Retrospective cohort	27 751	Women with a singleton pregnancy in cephalic presentation without gestational diabetes or preexisting diabetes or hypertension
Stock et al,^33^ 2012	Retrospective cohort	827 404	Women with a singleton pregnancy without preexisting disease or a previous adverse pregnancy outcome
Zenzmaier et al,^34^ 2021	Retrospective cohort	235 076	Women with a singleton pregnancy without indications for medical induction of labor

Abbreviation: BMI, body mass index (calculated as weight in kilograms divided by height in meters squared).

### Synthesis of Results

**Figure 2.**
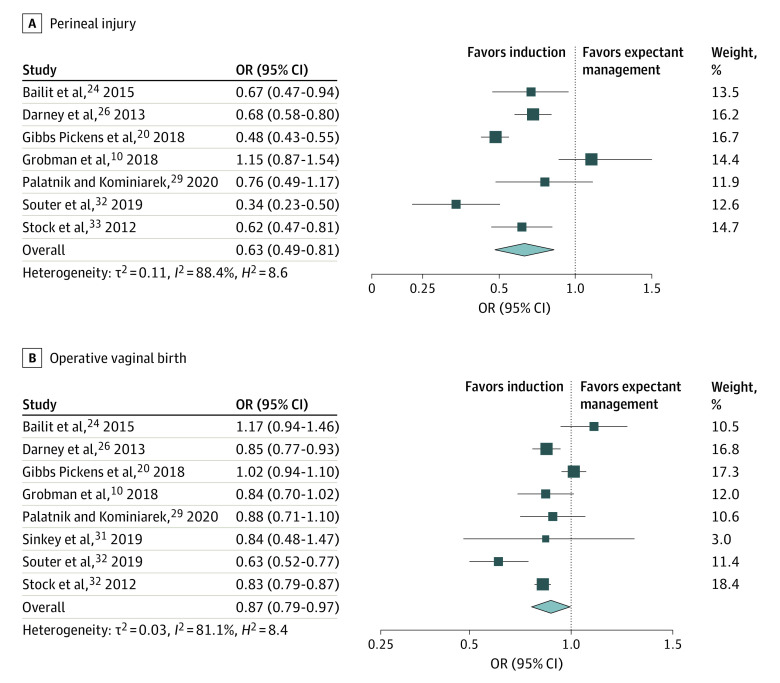
Perineal Injury and Operative Vaginal Birth Among Women Undergoing Elective Induction of Labor at 39 Weeks of Gestation Compared With Expectant Management Square size denotes weighting. The diamond represents the overall effect size. OR indicates odds ratio.

**Figure 3.**
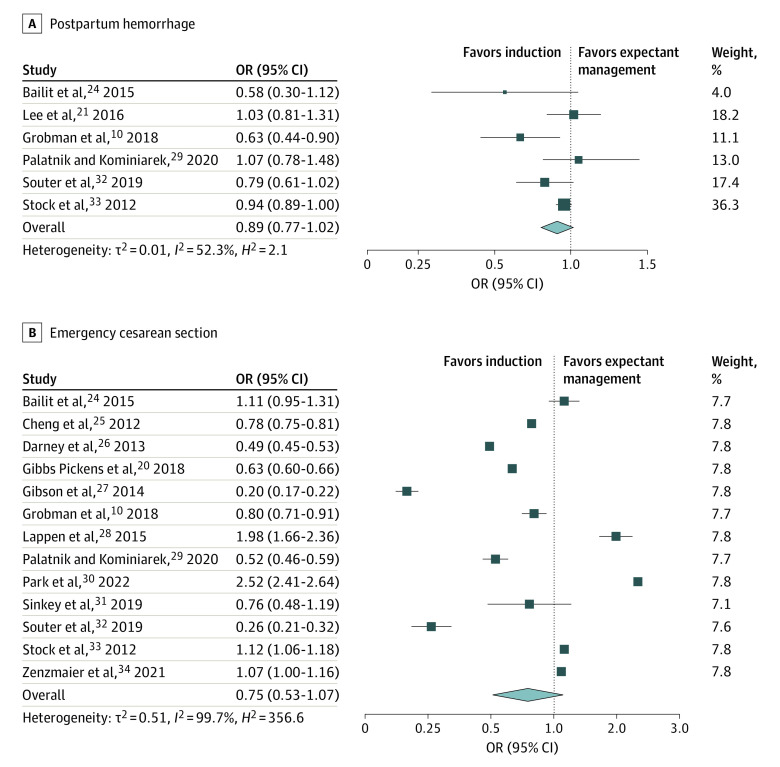
Postpartum Hemorrhage and Emergency Cesarean Section Among Women Undergoing Elective Induction of Labor at 39 Weeks of Gestation Compared With Expectant Management Square size denotes weighting. The diamond represents the overall effect size. OR indicates odds ratio.

Across the studies, 8 outcomes were commonly reported. These included the maternal outcomes of third- or fourth-degree perineal injury, operative vaginal birth, postpartum hemorrhage, and emergency cesarean section (Figures 2 and 3 and Table 2). Neonatal outcomes commonly reported were macrosomia, shoulder dystocia, NICU admission, and low 5-minute Apgar score (Table 2).

**Table 2.** Induction of Labor at 39 Weeks of Gestation vs Expectant Management

Outcome	No. of studies	Induction of labor, No./total No.	Expectant management, No./total No.	*I*^2^ (%)	Odds ratio (95% CI)^a^
Maternal					
Third- or fourth-degree perinatal injury	7	679/37 575	15 835/1 021 288	88.4	0.63 (0.49-0.81)
Operative vaginal birth	8	3598/45 209	130 357/1 146 394	88.1	0.87 (0.79-0.97)
Postpartum hemorrhage	6	1442/25 563	63 762/932 257	52.3	0.89 (0.77-1.02)
Emergency cesarean section	14	17 876/84 771	206 989/1 501 691	99.7	0.75 (0.53-1.07)
Neonatal					
Macrosomia	4	1081/23 146	24 196/295 524	93.4	0.66 (0.48-0.91)
Shoulder dystocia	5	633/30 078	6725/314 511	78.7	1.00 (0.91-1.08)
NICU admission	9	3392/48 773	74 534/1 039 309	96.8	0.84 (0.66-1.08)
Low Apgar score (<7 at 5 min after delivery)	4	117/21 828	931/97 989	63.5	0.62 (0.40-0.96)

Abbreviation: NICU, neonatal intensive care unit.

^a^ Calculated using a random-effects model and including all studies.

Compared with expectant management, elective induction of labor at 39 weeks of gestation was associated with a 37% reduced likelihood of third- or fourth-degree perineal injury (7 studies^10,20,24,26,29,32,33^; OR, 0.63 [95% CI, 0.49-0.81]) (Figure 2). Induction of labor was also associated with a reduced likelihood of operative vaginal birth (8 studies^10,20,24,26,29,31,32,33^; OR, 0.87 [95% CI, 0.79-0.97]) (Figure 2). Nonsignificant reductions in postpartum hemorrhage (6 studies^10,21,24,29,32,33^; OR, 0.89 [95% CI, 0.77-1.02]) and emergency cesarean section (14 studies^10,20,21,24,25,26,27,28,29,30,31,32,33,34^; OR, 0.75 [95% CI, 0.53-1.07]) were also observed (Figure 3 and Table 2).

For neonatal outcomes, elective induction was associated with a 34% reduced likelihood of macrosomia (4 studies^20,26,31,32^; OR, 0.66 [95% CI, 0.48-0.91]) and a 38% reduced likelihood of low 5-minute Apgar score (4 studies^10,25,29,32^; OR, 0.62 [95% CI, 0.40-0.96]). There was no difference between groups in the likelihood of shoulder dystocia (5 studies^20,26,27,31,32^; OR, 1.00 [95% CI, 0.91-1.08]) or NICU admission (9 studies^10,20,24,28,29,30,31,32,33^; OR, 0.84 [95% CI, 0.66-1.08] (Table 2).

Among multiparous women only (n = 336 303), induction of labor at 39 weeks of gestation (n = 27 670) was associated with a reduced likelihood of third- or fourth-degree perineal injury (OR, 0.73 [95% CI, 0.58-0.93]), emergency cesarean section (OR, 0.61 [95% CI, 0.38-0.98]), and macrosomia (OR, 0.69 [95% CI, 0.52-0.92). Nonsignificant reductions in shoulder dystocia (OR, 0.86 [95% CI, 0.71-1.04]) and NICU admission (OR, 0.78 [95% CI, 0.60-1.02]) were also observed. There were no differences between groups in the likelihood of operative vaginal birth (OR, 1.01 [95% CI, 0.84-1.21]), postpartum hemorrhage (OR, 0.93 [95% CI, 0.66-1.32]), or low 5-minute Apgar score (OR, 0.86 [95% CI, 0.53-1.38]) (Table 3).

**Table 3.** Adverse Outcomes Stratified by Parity

Outcome	Nulliparity (n = 407 302)					Multiparity (n = 336 303)				
	No. of studies	Induction of labor, No./total No.	Expectant management, No./total No.	*I* ^2^	Odds ratio (95% CI)	No. of studies	Induction of labor, No./total No.	Expectant management, No./total No.	*I* ^2^	Odds ratio (95% CI)
Maternal										
Third- or fourth-degree perineal injury	6	453/8423	9943/157 637	19.9	0.97 (0.85-1.10)	4	173/14 334	1507/120 423	35.7	0.73 (0.58-0.93)
Operative vaginal birth	6	1063/10 569	18 775/170 734	80.2	1.11 (0.93-1.33)	4	743/17 885	7001/154 691	79.3	1.01 (0.84-1.21)
Postpartum hemorrhage	5	189/4986	2462/72 160	0	0.93 (0.77-1.12)	3	107/4226	1302/49 036	59.4	0.93 (0.66-1.32)
Emergency cesarean section	10	10 209/31 444	108 570/358 554	95.4	0.80 (0.70-0.91)	6	1066/21 760	13 336/214 374	97.5	0.61 (0.38-0.98)
Neonatal										
Macrosomia	3	221/6345	10 346/142 314	93.1	0.65 (0.32-1.36)	3	837/16 358	13 610/151 053	90.9	0.69 (0.52-0.92)
Shoulder dystocia	4	127/7422	2308/147 185	0	1.22 (1.02-1.46)	4	485/22 206	4326/165 087	72.9	0.86 (0.71-1.04)
NICU admission	5	755/8605	8166/97 586	58.4	0.75 (0.63-0.89)	3	638/13 341	4965/84 318	77.1	0.78 (0.60-1.02)
Low Apgar score (<7 at 5 min after delivery)	4	97/18 112	747/68 096	45.6	0.65 (0.34-1.16)	2	20/3715	183/29 891	0	0.86 (0.53-1.38)

Abbreviation: NICU, neonatal intensive care unit.

Among nulliparous women only (n = 407 302), induction at 39 weeks of gestation (n = 31 947) was associated with a decreased likelihood of emergency cesarean section (9 studies^10,20,24,25,26,27,29,32,34^; OR, 0.80 [95% CI, 0.70-0.91]) and NICU admission (5 studies^10,20,24,29,32^; OR, 0.75 [95% CI, 0.63-0.89]). There was no difference between groups in terms of third- or fourth-degree perineal injury (OR, 0.97 [95% CI, 0.85-1.10]), operative vaginal birth (OR, 1.11 [95% CI, 0.93-1.33]), postpartum hemorrhage (OR, 0.93 [95% CI, 0.77-1.12]), or low 5-minute Apgar score (OR, 0.65 [95% CI, 0.34-1.16]). However, among this group, elective induction at 39 weeks of gestation was associated with an increased likelihood of shoulder dystocia (OR, 1.22 [95% CI, 1.02-1.46]) (Table 3).

### Risk of Bias

All observational studies^20,21,24,25,26,27,28,29,30,31,32,33,34^ assessed using the NOS were judged to be of good quality (scoring >3 stars in the selection domain, >3 stars in the comparability domain, and >2 stars in the outcome/exposure domain) (eTable 2 in Supplement 1). The single randomized trial^10^ included in our review was assessed as low risk of bias across all 5 domains of the Cochrane RoB 2 assessment.

## Discussion

The findings of this systematic review and meta-analysis suggest that compared with expectant management, elective induction of labor at 39 weeks of gestation was associated with a decreased likelihood of labor-related complications, including a 37% reduced likelihood of third- or fourth-degree perineal injury. Overall, induction of labor was also associated with a reduced likelihood of operative vaginal birth, macrosomia, and low 5-minute Apgar score. Results were similar when stratified by parity; however, both multiparous and nulliparous women had a reduced likelihood of emergency cesarean section. Among nulliparous women only, elective induction at 39 weeks was associated with an increased likelihood of shoulder dystocia. These findings suggest that compared with expectant management, elective induction of labor at 39 weeks of gestation is associated with lower rates of emergency cesarean section and of other labor-related and neonatal complications.

The practice shift toward elective induction at 39 weeks of gestation has been largely driven by the ARRIVE trial, which demonstrated a decreased risk of emergency cesarean birth with induction compared with expectant management.^10^ While subsequent meta-analyses have supported these findings,^11,35,36^ others have shown no association between induction of labor and cesarean section rates.^37,38,39,40^ Our findings are consistent with those of the ARRIVE trial, which demonstrated an 18% reduced likelihood of emergency cesarean section.

Previous meta-analyses examining outcomes following induction of labor at 39 weeks have largely focused on the primary outcome of emergency cesarean section, with limited investigation of other labor-related outcomes. In keeping with our study, a 2020 meta-analysis by Middleton et al^12^ found that compared with expectant management, induction of labor was associated with a reduced risk of cesarean section and low 5-minute Apgar score. However, in contrast with our findings, Middleton et al^12^ reported no difference in other birthing outcomes including perineal injury, operative vaginal birth, and postpartum hemorrhage. A critical difference between the meta-analysis by Middleton et al^12^ and ours is that the former study only included randomized clinical trials and examined outcomes following induction of labor from 37 weeks of gestation rather than induction at 39 weeks only. We chose 39 weeks of gestation because we consider it more clinically relevant: induction of labor without medical indication at 37 weeks of gestation should be discouraged.^41^

To investigate the impact of induction of labor at 39 weeks outside of a randomized trial setting, Grobman and Caughey^11^ performed a meta-analysis including only cohort studies. The primary outcome was cesarean section, which they found was reduced with induction of labor compared with expectant management. They also reported a significant reduction in the risk of peripartum infection and a potential trend toward reduced third- and fourth-degree perineal injury (risk ratio, 0.91 [95% CI, 0.78-1.07]) and postpartum hemorrhage (risk ratio, 0.87 [95% CI, 0.54-1.41]).^11^ Although nonsignificant, these trends support our findings of a reduced likelihood of perineal injury and a potential reduction in postpartum hemorrhage.

Stratifying our analysis by parity, the results for multiparous women were comparable with those for the total population. However, induction of labor was also associated with a reduced likelihood of emergency cesarean section and macrosomia. Interestingly, nulliparous women were more likely to have a birth complicated by shoulder dystocia following induction of labor. To date, this finding has not been reported in previous meta-analyses and an explanation for it is not immediately clear.

To our knowledge, this is the largest systematic review and meta-analysis to examine maternal and neonatal complications following elective induction of labor at 39 weeks of gestation compared with expectant management. Our findings are largely reassuring. Among the 1 625 899 women included in the 14 studies reviewed, induction of labor at 39 weeks was associated with reduced maternal and neonatal complications. This provides further evidence that suggests the safety of induction of labor at 39 weeks. Importantly, these results may be applicable to a broader obstetric population, given the inclusion of both nulliparous and multiparous women, individuals with a BMI greater than 30, and women undergoing a trial of labor after a previous cesarean section. We examined differences between induction of labor and expectant management more holistically, investigating important maternal and neonatal outcomes that may contribute to shared decision making between clinicians and patients. We also demonstrated an important difference between nulliparous and multiparous women, and these results should be used to provide more personalized evidence to women considering induction of labor.

### Limitations

This systematic review and meta-analysis was limited by the small number of observational studies and relevant randomized trials. Given that the majority of included studies were observational, our results may have been affected by classification biases, specifically misclassification of the outcomes and potentially underreporting. Moreover, these observational studies may also have been affected by uncontrolled or unmeasured confounding factors. Thus, further randomized clinical trials are needed to strengthen the existing evidence base in this setting.

## Conclusions

This review of 1 625 899 women from 14 studies found that elective induction of labor at 39 weeks of gestation compared with expectant management was associated with improved labor-related outcomes, including a 37% reduction in perineal injury risk. Our findings suggest that elective induction of labor at 39 weeks of gestation is likely to be safe and beneficial for some women, but the benefits must be weighed against the potential increased risks of shoulder dystocia among nulliparous individuals.

## Notes

Supplement 1. **eTable 1.** Included Search Terms **eTable 2.** Newcastle-Ottawa Quality Assessment Scale for Nonrandomized Studies

Supplement 2.
Data Sharing Statement
