# Supplementary material for: Comparison of Maternal Labor-Related Complications and Neonatal Outcomes Following Elective Induction of Labor at 39 Weeks of Gestation vs Expectant Management: A Systematic Review and Meta-analysis
Source: JAMA Netw Open. 2023 May 12;6(5):e2313162. doi: 10.1001/jamanetworkopen.2023.13162 (PMC10182428; doi:10.1001/jamanetworkopen.2023.13162)
Supplement: Supplement 1. — eTable 1. Included Search Terms eTable 2. Newcastle-Ottawa Quality Assessment Scale for Nonrandomized Studies [file jamanetwopen-e2313162-s001.pdf]

Supplementary Online Content

Hong J, Atkinson J, Roddy Mitchell A, et al. Comparison of maternal labor-related complications and neonatal outcomes following elective induction of labor at 39 weeks of gestation vs expectant management: a systematic review and meta-analysis. *JAMA Netw Open*. 2023;6(5):e2313162. doi:10.1001/jamanetworkopen.2023.13162

eTable 1. Included Search Terms

eTable 2. Newcastle-Ottawa Quality Assessment Scale for Nonrandomized Studies

This supplementary material has been provided by the authors to give readers additional information about their work.

© 2023 Hong J et al. *JAMA Network Open*.

eTable 1. Included Search Terms

1	labour.mp.
2	induction.mp.
3	induction of labour.mp.
4	perineal injury.mp.
5	Perineum {Including Related Terms}
6	postpartum haemorrhage.mp.
7	obstetric labor complications.mp.
8	Intensive Care Units, Neonatal {Including Related Terms}
9	anal sphincter injury {Including Related Terms}
10	shoulder dystocia {Including Related Terms}
11	macrosomia {Including Related Terms}
12	3rd degree tear {Including Related Terms}
13	4th degree tear {Including Related Terms}

© 2023 Hong J et al. *JAMA Network Open*.

eTable 2. Newcastle-Ottawa Quality Assessment Scale for Nonrandomized Studies

[a] Determined based on thresholds for converting the Newcastle-Ottawa scales to Agency for Healthcare Research and Quality (AHRQ). Good quality: 3 or 4 stars in selection domain and 1 or 2 stars in comparability domain and 2 or 3 stars in outcome/exposure domain. Fair quality: 2 stars in selection domain and 1 or 2 stars in comparability domain and 2 or 3 stars in outcome/exposure domain. Poor quality: 0 or 1 star in selection domain or 0 stars in comparability domain or 0 or 1 star in outcome/exposure domain.

Source	Selection (/4)	Comparability (/2)	Outcome (/3)	Overall study quality[a]
**Bailit et al, 2015**	4	2	2	Good
**Cheng et al, 2012**	4	2	2	Good
**Darney et al, 2013**	4	2	3	Good
**Gibbs-Pickens et al, 2018**	4	2	3	Good
**Gibson et al, 2014**	4	2	2	Good
**Lappen et al, 2015**	4	2	2	Good
**Lee et al, 2016**	4	2	3	Good
**Palatnik et al, 2020**	4	2	2	Good
**Park et al, 2020**	3	2	2	Good
**Sinkey et al, 2019**	4	2	2	Good
**Souter et al, 2019**	4	2	2	Good
**Stock et al, 2012**	4	2	2	Good
**Zenzmaier et al, 2020**	4	2	3	Good

© 2023 Hong J et al. *JAMA Network Open*.
