# Supplementary material for: Comparison of Maternal Labor-Related Complications and Neonatal Outcomes Following Elective Induction of Labor at 39 Weeks of Gestation vs Expectant Management: A Systematic Review and Meta-analysis
Source: JAMA Netw Open. 2023 May 12;6(5):e2313162. doi: 10.1001/jamanetworkopen.2023.13162 (PMC10182428; doi:10.1001/jamanetworkopen.2023.13162)
Supplement: Supplement 2. — Data Sharing Statement [file jamanetwopen-e2313162-s002.pdf]

Data Sharing Statement

Hong. Comparison of Maternal Labor-Related Complications and Neonatal Outcomes Following Elective Induction of Labor at 39 Weeks of Gestation vs Expectant Management. *JAMA Netw Open*. Published May 12, 2023. doi:10.1001/jamanetworkopen.2023.13162

Data
Data available: No

Additional Information
Explanation for why data not available: No participant data collected for this manuscript. All data will be available within manuscript and has been collected by the respective authors of papers included in the meta-analysis.
